# Percutaneous balloon mitral valvuloplasty: is there still a place for it in the Netherlands?

**DOI:** 10.1007/s12471-019-01343-7

**Published:** 2019-10-25

**Authors:** P. den Heijer

**Affiliations:** Heart Center, Amphia Hospital, Breda, The Netherlands

In this issue of the Netherlands Heart Journal, Ambari et al. present a survival analysis of patients with rheumatic mitral stenosis after percutaneous balloon mitral valvuloplasty (PBMV) compared with mitral valve surgery in a low-to-middle income country [1]. From their and other studies it can be concluded that PBMV is a reasonable alternative to mitral valve surgery, offering a comparable survival prognosis, albeit, as expected, with a considerably lower sustained event-free duration. The relatively high recurrence rate of mitral stenosis is such, that the main motive for offering PBMV to patients suffering from rheumatic mitral stenosis must be its low cost; PBMV is much cheaper than cardiac surgery. Over the years, PBMV has flourished as a mainstream therapy, particularly in developing countries with endemic rheumatic heart disease. Once I met a prominent Indian interventional cardiologist, who mentioned that his average patient load for PBMV was 40 patients. “A week?”, I asked. No, he performed 40 procedures a day!

So, what about PBMV in the Netherlands? Dutch interventional cardiologists have always been eager to be on the front line of innovation and embrace new techniques as soon as they become available. In the eighties, we were among the first in Europe to follow Cribier with aortic balloon valvuloplasty [2, 3]. And shortly after the first percutaneous mitral valvuloplasty’s by pioneers such as Babic, Vahanian and Block [4–6], PBMV programmes were initiated in Amsterdam, Eindhoven, Nieuwegein and Groningen. These programmes were modest in volume and somewhat hampered by suboptimal catheter materials and difficult techniques, such as double-balloon inflations or the combination of an anterograde transseptal approach and a retrograde wire loop approach. Only a few years later, a breakthrough technology came over from Japan: the brilliantly designed Inoue-balloon catheter. With this catheter, transseptal PBMV became an operator-friendly, easy-to-perform procedure overnight [7]. Now, 35 years later, and as mentioned by Ambari et al., the Inoue-balloon catheter remains the standard for PBMV.

In the Netherlands, PBMV became an accepted procedure, but over the years it remained low-volume compared with mitral valve surgery. Not only did our low incidence of rheumatic valve disease limit further expansion but also our use of a clear scoring system. This scoring system, predicting safety and success, guided operators to better—more restrictively—select their patients [7]. Moreover, cardiothoracic surgeons raised their skills and techniques to very high levels with outstanding results and started using minimally invasive, port-access techniques for mitral valve replacement and repair [8].

A quick survey learned that seven Dutch heart centres still perform PBMV today, albeit occasionally, with a total of around 20 procedures per year in the Netherlands. These procedures are invariably done by interventional cardiologists with long experience in much more complex structural interventions such as transcatheter aortic valve implantation and MitraClip. With perhaps the exception of the sporadic young female patient, presenting with a true rheumatic, non-calcified mitral stenosis with fused commissures and a reluctancy to take oral anticoagulants, most patients have severe illness and co-morbidity. Thus, PBMV in the Netherlands has typically become a last-resort treatment for inoperable patients.

In the developing countries, PBMV is of immense value for health care, saving numerous lives. In the Netherlands, PBMV still exists as an occasionally useful therapy, but its place is very limited.
